# National lung cancer screening program feasibility study in Estonia

**DOI:** 10.1093/icvts/ivad041

**Published:** 2023-02-21

**Authors:** Kadi Kallavus, Kaja-Triin Laisaar, Anneli Rätsep, Tarvo Kiudma, Urmas Takker, Anneli Poola, Vahur Makke, Marianna Frik, Piret Viiklepp, Merily Taur, Tanel Laisaar

**Affiliations:** Institute of Clinical Medicine, University of Tartu, Tartu, Estonia; Institute of Family Medicine and Public Health, University of Tartu, Tartu, Estonia; Institute of Family Medicine and Public Health, University of Tartu, Tartu, Estonia; Institute of Family Medicine and Public Health, University of Tartu, Tartu, Estonia; Ränilinna Health Centre, Tartu, Estonia; Puusepa Health Centre, Tartu, Estonia; Family Physicians Takker and Sarapuu, Tartu, Estonia; Radiology Clinic, Tartu University Hospital, Tartu, Estonia; Radiology Clinic, Tartu University Hospital, Tartu, Estonia; Radiology Clinic, Tartu University Hospital, Tartu, Estonia; Head of Department of Registries, National Institute for Health Development, Tallinn, Estonia; Lung Clinic, Tartu University Hospital, Tartu, Estonia; Institute of Clinical Medicine, University of Tartu, Tartu, Estonia; Lung Clinic, Tartu University Hospital, Tartu, Estonia

**Keywords:** Lung cancer, Screening, Low-dose computed tomography, Feasibility

## Abstract

**OBJECTIVES:**

The main aim of the lung cancer screening (LCS) feasibility study was to investigate the plausibility of and bottlenecks to systematic enrolment in family physician practices by evaluating all their patients.

**METHODS:**

In 3 family physician practices, for each individual born in 1947–1966 (target age group 55–74 years), information on ever smoking was gathered by a family physician/nurse. All current and ex-smokers were invited to an ‘LCS visit’. In parallel, 2 inclusion criteria were used: (1) current smoker (≥20 pack-years) or ex-smoker (quit <15 years ago and smoking history ≥20 pack-years) and (2) PLCO_m2012noRace_ risk score >1.5. All individuals with elevated lung cancer risk were assigned low-dose computed tomography.

**RESULTS:**

Among the total 7035 individuals in the 3 family physician practices, the LCS target age group comprised 1208 individuals, including 649 (46.3–57.1%) males and 559 (42.9–53.7%) females. Of the 1208 applicable age group individuals, 395 (all current or ex-smokers) were invited to the ‘LCS visit’. According to either 1 or both the LCS inclusion criteria, 206 individuals were referred to low-dose computed tomography, and 201 (97.6% of those referred) ended up taking it. The estimated participation rate in LCS, based on data from our feasibility study, would have been 87.4%.

**CONCLUSIONS:**

In LCS, systematic enrolment of individuals by family physicians results in high uptake, and thus, effectiveness of the LCS in the setting of a well-functioning family physician system like in Estonia. Also, the feasibility study provided excellent input to the currently ongoing regional LCS pilot study in Estonia.

**Figure:**
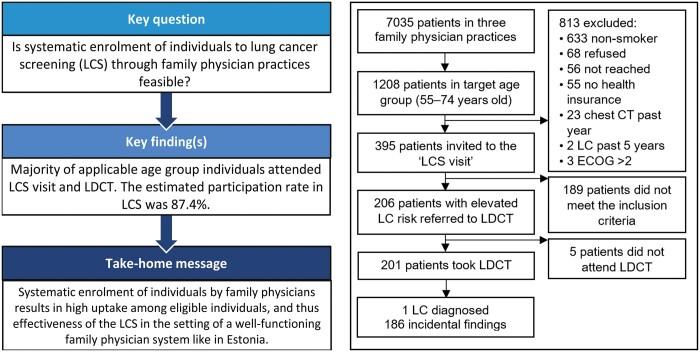

## INTRODUCTION

Lung cancer screening (LCS), using low-dose computed tomography (LDCT), has been proved to reduce mortality in 2 large, randomized controlled studies: National Lung Cancer Screening Trial (NLST) in the USA and Dutch-Belgian Randomized Lung Cancer Screening Trial (Dutch acronym: NELSON) in Europe [1, 2]. Implementation of LCS is currently recommended by many international medical societies [3, 4] and supported by several guidelines [5–7]. However, no consensus on who and exactly how should be screened has been reached yet [8]. Several ongoing studies are evaluating these open issues. Among others, one of the biggest challenges remains how to assure high participation of eligible participants in an LCS program.

LCS pilot studies are being conducted, and regional or national programs initiated in many European countries, e.g. Czech Republic [9] and Poland [10]. Other countries are at a various stages of LCS implementation [8]. In Estonia, a decision was made to implement LCS stepwise, starting with a feasibility study and, after having gained necessary information, proceed with a regional pilot study, involving about 10% of the total population of the country (which is currently ongoing).

Estonia has a centralized health system with a single health insurance fund. Primary care acts as the first point of contact in healthcare. Each insured person has his/her own family physician, and visits to the family physician are free of charge. In Estonia, about 94% of the population has health insurance [11]. According to an annual population-based survey, 68% of the population has visited a family physician practice in past 12 months [12]. Thus, majority of individuals have close contact with family physician, which allows systematic enrolment of individuals through their practices and provides excellent overview of participation in LCS.

The main aim of the feasibility study, described in the current study, was to test a systematic approach of enrolling individuals through family physicians reviewing and evaluating all their patients.

## METHODS

### Ethics statement

The study was approved by the Research Ethics Committee of the University of Tartu (337/T-17; 15 March 2021). Written informed consent was signed by study participants.

### Study design and participants

In a family physician practice, for each individual born in 1947–1966 (as for LCS the target age group was 55–74 years) information on whether he/she had ever smoked was gathered by a family physician or nurse. This information was obtained from either patient’s case report, during an out-patient visit performed due to any reason or in a short phone interview. In case the information was available, the following exclusion criteria were considered:

- non-smoker (having smoked <100 cigarettes in lifetime),
- no health insurance,
- lung cancer (LC) diagnosis during past 5 years,
- chest computed tomography (CT) scan performed during past year and/or
- poor performance status, precluding cancer treatment (eastern cooperative oncology group (ECOG) >2) [13].

Also, individuals refusing from any cooperation or not reached after several attempts were excluded.

All current and ex-smokers, irrespective of their smoking duration and intensity, and without any of the above-defined exclusion criteria were invited to an ‘LCS visit’. During this visit, each individual was provided LCS (in general) and the feasibility study details. After the individual had signed a written informed consent, his/her LCS feasibility study exclusion criteria were re-evaluated, smoking history was detailed and LC risk score PLCO_m2012noRace_ value was calculated [14]. The PLCO_m2012noRace_ (https://brocku.ca/lung-cancer-screening-and-risk-prediction/risk-calculators/) model predicts incident LCs within 6 years of screening using the following predictors: age, education, body mass index, history of chronic obstructive pulmonary disease (COPD), personal history of cancer, family history of LC, smoking status, intensity (mean number of cigarettes smoked per day), duration, and quit-years in individuals who used to smoke [14].

We used 2 potential inclusion criteria in parallel—smoking status (in addition to age) and LC risk score. Individuals meeting either 1 or both the following inclusion criteria were enrolled into the study:

- current smoker with smoking duration ≥20 pack-years or ex-smoker, who quit <15 years ago and had a smoking history ≥20 pack-years and
- PLCO_m2012noRace_ risk score >1.5.

The 2 inclusion criteria were used mainly to test the applicability and complexity of LC risk score use by family physicians and nurses, in comparison to the more simple approach based on individual’s age and smoking status. Smoking criteria were selected based on current recommendations of international societies [3, 5, 7]. PLCO risk score is most widely used in currently ongoing LCS trials and has demonstrated to have the best predictive value [14–16].

All individuals with elevated LC risk were assigned LDCT. In case the individual did not appear to the assigned LDCT appointment, according to the study protocol a study coordinator called him/her and provided a new appointment (time). In case the individual failed to appear to this second appointment, he/she was considered a drop-out and excluded from the study.

LDCTs were evaluated independently by 2 radiologists and described according to LungRADS version 1.1 protocol [17]. Further management of indivduals followed the recommendations of the LungRADS protocol. In addition to lung nodules, all pulmonary and extrapulmonary findings were outlined. Individuals with pulmonary findings were further evaluated by a study pneumologist, who paid special attention to lung emphysema in order to define individuals requiring further evaluation for undiagnosed COPD. Extrapulmonary findings were managed by family physicians according to common practice.

Inclusion of individuals into the study was planned for a period of 7 months (from 1 May 2021 to 30 November 2021), meaning that each family physician/nurse should have evaluated ∼15% of all his/her patients each month to ensure enrolment of all (eligible) patients.

For the LCS feasibility study, an online database, using the REDCap platform in the University of Tartu secure server, was created.

## RESULTS

### Enrolment and participation

The 3 family physician practices had in total 7035 (per practice 2150–2589) patients. The LCS target age group (55–74 years old) formed 13.3–20.0% of all the patients in these practices—altogether 1208 individuals, with 649 (46.3–57.1%) males and 559 (42.9–53.7%) females.

Of the 1208 applicable age group individuals, 813 had 1 or more exclusion criteria:

- non-smoker—633 (77.9%),
- refused to participate—68 (8.4%),
- not reached—56 (6.9%),
- no health insurance—55 (6.8%),
- chest CT performed in the past year—23 (2.8%),
- LC diagnosis in past 5 years—2 (0.2%) and/or
- poor performance status—3 (0.4%).

In total, 395 individuals (5.6% of all the patients in these 3 family physician practices and 32.9% of the total target age group in the practices) were invited to the ‘LCS visit’.

During the ‘LCS visit’ each individual’s smoking status was detailed and his/her LC risk score was calculated. Altogether, in the 3 family physician practices:

- 198 individuals (16.4% of the target age group) had an elevated LC risk according to the smoking criteria, including 134 current and 64 ex-smokers, and
- 125 individuals (10.3% of the target age group) had an elevated LC risk according to the LC risk score PLCO_m2012noRace_.

Altogether 206 individuals had an elevated LC risk according to either 1 or both the LCS inclusion criteria and were referred to LDCT. Of them, 117 met both the inclusion criteria, 81 met only the smoking criteria (in addition to age) and 8 only had an elevated LC risk score.

**Figure 1:**
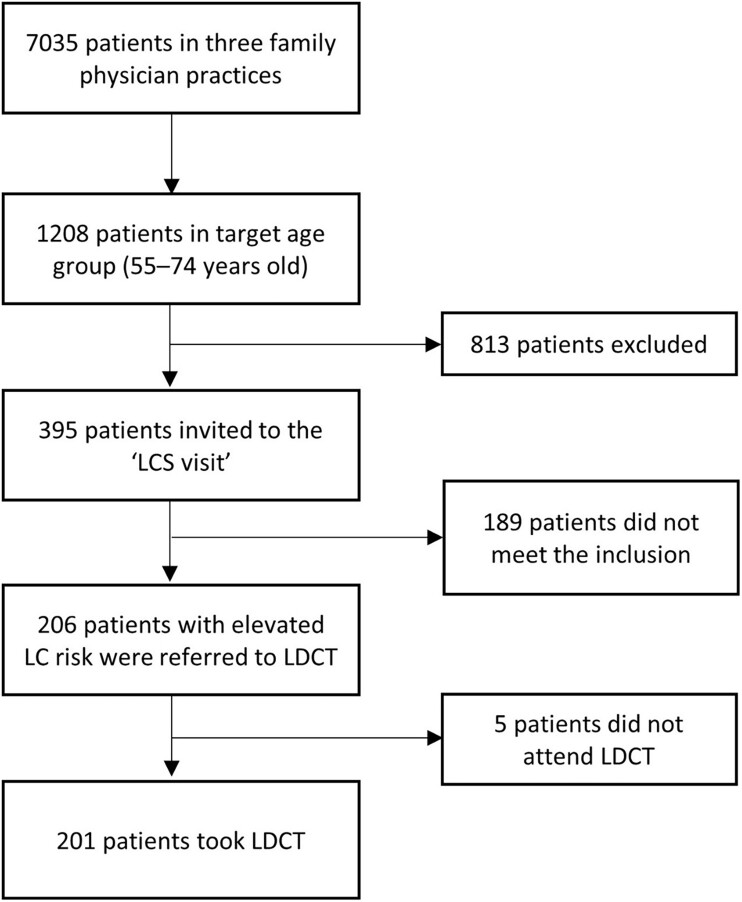
Estonian lung cancer screening feasibility study participant recruitment flowchart.

Out of the 206 individuals referred, 201 ended up taking LDCT (97.6% of individuals referred to LDCT, 16.6% of the total target age group and 2.9% of all 3 family physician practices patients). Of the 201 individuals taking LDCT, 192 (95.5%) showed up for CT at their first allocated time and 9 (4.5%) for the second appointment (Fig. 1).

**Figure 2:**
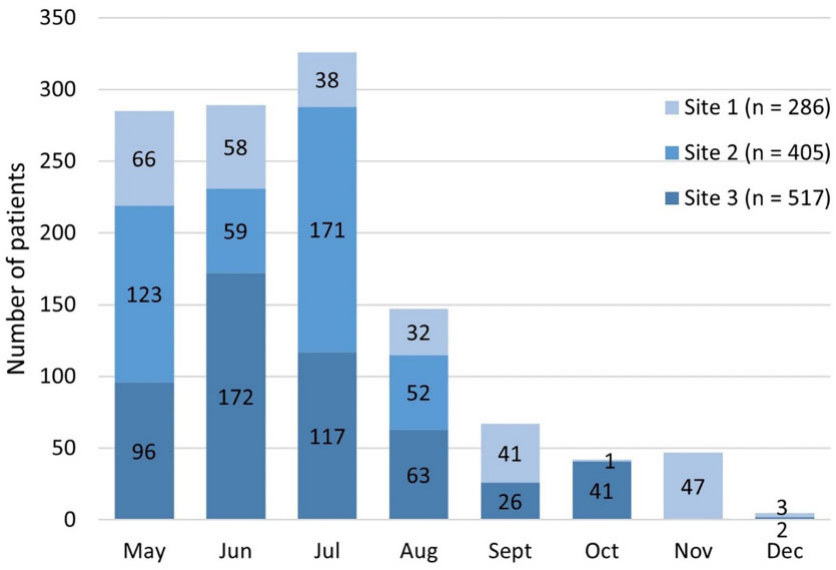
Inclusion of target age group patients into the Estonian lung cancer screening feasibility study.

Although inclusion into the LCS feasibility study was planned for the period of 7 months, it was completed considerably faster in all 3 family physician practices (Fig. 2). Majority (75%) of the target age group individuals were evaluated within the first 3 months of the study.

### Lung cancer screening results

In the LCS feasibility study, 1 case of LC was detected, and 27 (13.4%) individuals needed a repeat CT after 3 or 6 months due to the finding of an indeterminate pulmonary nodule. None of the nodules demonstrated growth on follow-up CT-s.

In addition to lung nodules, 186 (92.5%) individuals had other incidental pulmonary or extrapulmonary findings:

- coronary artery calcifications—120 individuals (59.7%),
- lung emphysema—115 (57.2%),
- bronchiectasis—2 (1.0%),
- mediastinal lymphadenopathy—1 (0.5%),
- kidney stones—7 (3.5%),
- suprarenal adenoma—4 (2.0%),
- kidney cancer—1 (0.5%) and
- subscapular elastofibroma—1 (0.5%).

### Estimated lung cancer screening target group size and participation rate

In the 3 family physician practices, 1208 individuals fell into the LCS target age group of 55–74 years. However, 124 of them were excluded from the study due to refusal or could not be reached, and thus, information on their smoking status and LC risk score remained unknown. From the included 1084 individuals, 206 (19%) had elevated LC risk and were referred to LDCT. Assuming that the same proportion (19%) of those who refused or were not reached would have had elevated LC risk, 24 more individuals should have been referred to LDCT. Based on this assumption, from all the target age group individuals in these 3 family physician practices, for 230 LCS with LDCT would have been indicated. As in reality 201 individuals reached LDCT, the estimated participation rate in LCS in our feasibility study was 87.4%.

## DISCUSSION

LC is the leading cause of cancer-related deaths worldwide [18]. Approximately 70% of patients have advanced disease (stage III/IV) at the time of diagnosis, and thus survival remains poor [19]. The overall survival at stage IV LC is 10% compared to about 80% at stage I [19]. LDCT reduces LC mortality in high-risk individuals through early detection, as 2 large randomized controlled trials—NLST and NELSON—have demonstrated [1, 2]. Moreover, the larger of these 2, NLST, also showed a significant all-cause mortality reduction of 6.7% [1].

To be effective, LCS requires targeting of individuals most at risk. In this regard, LCS differs from other population-based screening programs such as breast, cervical and bowel cancers, where all individuals of certain age and sex are eligible. In LCS, different methods are used for identifying people who are at high risk for LC, and thus potentially eligible for screening. In 2 studies conducted in the UK, Lung Screen Uptake Trial [20] and Yorkshire Lung Screening Trial (YLST) [16], it was possible to preselect ever smokers from primary care records, but many countries have not established a national primary care database, and even less have a database that includes individuals’ smoking status. In most LCS clinical trials, population-based or primary care registers are utilized to select individuals, and direct mailing is used as the recruitment strategy [2, 20, 21]. In some studies, mass media advertisements or community outreach has been added [22, 23]. Research papers provide limited description of how the primary care registers were reviewed, including how systematic the review process was.

In the Estonian LCS feasibility study, initial information about smoking was gathered by a family physician/nurse either from patient’s case report, during an out-patient visit performed due to any reason during the recruitment period or in a short phone interview. Systematically going through the whole list of patients in each family physician practice allows accurate tracking of all applicable individuals (for LCS with LDCT). This approach enables to estimate the participation rate, define the steps of inclusion and analyse the characteristics of excluded individuals or drop-outs. Although mass media and community awareness activities have a wide audience reach, they are ineffective in attracting all eligible individuals [24]. Moreover, inclusion bias is likely to occur. In contrast, primary care invitations have shown to increase the effectiveness of screening [15], as also demonstrated in our LCS feasibility study.

In the Estonian LCS feasibility study, almost 1/3 (32.9%) of the applicable age group patients were invited to a ‘LCS visit’, and majority of them attended it. Altogether half (52,2%) of the attended individuals had elevated LC risk and were referred to LDCT, with 97.6% of them ending up taking it. In the feasibility study, enrolling participants through family physicians ensured very high participation rates, both at the ‘LCS visit’ and LDCT stages. Our results are somewhat similar to the YLST [16], where primary care records were used to preselect ever smokers, and individuals were contacted by primary care physicians. In the YLST, 34.4% were eligible for a screening visit, and 99.7% had a baseline LDCT scan [16]. In the Lung Screen Uptake Trial, where all potential participants, algorithmically selected from primary care databases, were assessed for suitability by their general practitioner before the invitation was sent, 84.5% of participants were eligible for LDCT screening, with 91% choosing to be screened [20]. The UK Lung Cancer Screening (UKLS) pilot trial, where primary care records were used to identify eligible age group individuals, demonstrated a lower participation rate: 11.5% of participants were at high enough risk for trial entry; yet, only 46.5% of them gave their consent, of whom 49.1% underwent LDCT [25]. In the NELSON, where a population approach for only men was initially used, 24.9% of eligible men responded to a questionnaire, and 20.5% of the respondents met the eligibility criteria, of whom 24.4% were screened [2].

In conclusion, we can say that, considering the estimated participation rate of 87.4% in the total LCS target group (based on age and elevated LC risk), we were able to demonstrate that excellent LDCT coverage can be achieved with a systematic family physician-based approach.

In the Estonian LCS feasibility study, 2/3 (67%) of individuals in the applicable age group had to be excluded, with the main reason being a non-smoker (78% of the excluded individuals). The proportion of excluded persons in Estonia is similar to other LCS feasibility studies. It is important to note that in our study only 8% of individuals refused to participate, which is a considerably smaller proportion than reported in previous research [26, 27]. Our hypothesis is that the low refusal rate was achieved due to good doctor–patient relationship. According to the annual population-based survey, vast majority (85%) of individuals in Estonia were satisfied with their last family physician visit, and 83% of Estonian residents are convinced that their family physician can help them with most health issues [12].

Persons without health insurance, i.e. those never assigned to a family physician and those currently without health insurance, were excluded from the Estonian feasibility study but will probably be included in the national LCS program in the future. One could speculate that this would further increase the participation rate, as people with limited access to medical care would likely use the opportunity provided in the LCS program.

In our study, we tested the challenges and advantages of 2 potential inclusion criteria, the smoking status (in addition to age) and the LC risk score PLCO_m2012noRace_. Although calculating the LC risk score requires more time, according to our experience, i.e. feedback from family physicians and nurses who participated in the feasibility study, it was considered easy and did not hinder the inclusion process. To date, no studies have analysed physicians’ barriers associated with different inclusion criteria. However, studies have shown that physician’s (in)ability to manage time or competing priorities is barrier to communicating with patients [28]. In our study, we received similar feedback from the physicians and nurses, although they did the inclusion work in high speed—approached 75% of their target age group individuals within the first 3 months of the 7-month inclusion period.

In this LCS feasibility study, 1 case of LC was detected (corresponding to 0.5% of all the LDCTs performed), and 13.4% of LDCT individuals needed follow-up due to an indeterminate pulmonary nodule (according to the LungRADS protocol). In other feasibility studies and pilot trials, LC detection rate has varied between 0.4 and 2.0% [26, 27, 29]. In the NELSON, at baseline, the LC detection rate was 0.9%, and 19.7% of the baseline tests were initially indeterminate and these individuals underwent at least 1 additional CT scan [2]. In the Estonian LCS feasibility study, individuals with emphysema were analysed in more detail, as it is a disease with the same main risk factor (smoking), and as COPD is known to be underdiagnosed in Estonia. Emphysema was described in 115 individuals (57.2% of those who underwent the LDCT), and after additional investigations and evaluation by pneumologist, 8 new COPD cases were diagnosed (7.0% of individuals who underwent the LDCT).

To date, there is no consensus on describing and managing incidental findings in LCS. Excessive investigation and overreporting of incidental findings can cause anxiety, also damage due to unnecessary testing, and increase health system costs [8]. Development of an optimal protocol for managing incidental findings and adherence to the protocol can keep primary care workload and costs for the management of incidental findings under control [30].

### Limitations

Certain limitations must be taken into account when interpreting the results of the Estonian LCS feasibility study. The methodology used in this study, where people were enrolled through family physician/nurse, is applicable to countries where there is a similar well-developed primary healthcare system and majority of people have health insurance and a family physician. The 3 family physicians in this study had academic background and previous experience in clinical studies and could have thus, following their high professional standards, had higher motivation to take part in and complete the study. The study was conducted in a relatively small town (with population of about 100,000 inhabitants) with good access to medical care, including CT. And the results are based on only 3 family physician practices. However, this was the first step towards a national LCS program that provided valuable information for planning the currently ongoing regional LCS pilot study, where 10% of the population of the Estonia and also 10% of the family physician practices in the country are included. Although additional barriers to enrolment were short study period (7 months instead of a full year), and the COVID pandemic, these actually did not turn out to be real limitations, as 3/4 of the eligible age group individuals were reached during the first 3 months of the study.

Due to resource constraints, smoking cessation counselling was not mandatory in this feasibility study. However, all individuals were advised to quit smoking. Special smoking cessation counselling was offered when available.

## CONCLUSIONS

In LCS, systematic enrolment of participants by family physicians resulted in high uptake among eligible individuals and thus effectiveness of this population-based approach. Also, the feasibility study provided excellent input to the currently ongoing regional LCS pilot study in Estonia.

## Data Availability

The data underlying this article will be shared on reasonable request to the corresponding author.

## Notes

Presented at the 30th European Conference on General Thoracic Surgery (The Hague, Netherlands, World Forum Convention Centre, 19–21 June 2022), 20 June 2022.

## ABBREVIATIONS

COPD: Chronic obstructive pulmonary disease
LC: Lung cancer
LCS: Lung cancer screening
LDCT: Low-dose computed tomography
NELSON: Dutch-Belgian Randomized Lung Cancer Screening Trial
NLST: National Lung Cancer Screening Trial
YLST: Yorkshire Lung Screening Trial
